# Dietary diversity and its association with nutritional status among preschool children aged 2–5 years attending outpatient clinics in Karachi: A cross sectional study

**DOI:** 10.12669/pjms.42.(ICON26).15703

**Published:** 2026-04

**Authors:** Unaiza Khalid, Hiba Ashraf, Abdul Rehman

**Affiliations:** 1 Unaiza Khalid, Resident Family Medicine, FCPS. Department of Family Medicine, The Indus Hospital and Health Network, Korangi Crossing, Karachi, Pakistan; 2 Hiba Ashraf, FCPS. Department of Family Medicine, The Indus Hospital and Health Network, Korangi Crossing, Karachi, Pakistan; 3 Abdul Rehman, Research Associate, Office of Research, Innovation and Commercialization (O.R.I.C), The Indus Hospital and Health Network, Korangi Crossing, Karachi, Pakistan

**Keywords:** BMI, Dietary Diversity, Preschool children, Malnutrition, MUAC, Nutritional status

## Abstract

**Objective:**

To assess the dietary diversity among pre-school children aged 2-5 years visiting Family Medicine and Pediatric outpatient clinics in Karachi and compare it with their BMIs and MUACs.

**Methodology:**

This cross-sectional study was conducted in Family Medicine and Paediatric outpatient clinics of Indus Hospital Karachi from August 2024 till November 2024. A total of three hundred and thirty four children were included in the survey according to the inclusion criteria through non-probability consecutive sampling. The sample size was achieved six months after the approval of IRB. Data was collected using the Food Frequency Questionnaire and child’s socio demographic details which was then analysed on SPSS version 26 and compared with their BMI and MUACs in the end.

**Results:**

The majority of the preschool-aged children in the study were male, lived in cities, and had parents with poor incomes and little education. Of all the children, 81.4% had inadequate dietary diversity score (DDS), with a mean DDS of 2.46 ± 0.74. In addition to being positively correlated with BMI (β = 0.38, p = 0.025) and MUAC (β = 0.68, p < 0.001), higher DDS was also linked to older age, urban residency, and higher parental income and education.

**Conclusion:**

This study highlighted the major food group preferences among pre-school children aged 2-5 years, hence identifying gaps to guide about early interventions for improving child nutrition.

## INTRODUCTION

Nutritional status is the main key for determining health and well-being of an individual which ultimately improves his quality of life.^1^ Children are in a phase of rapid development hence their requirement is even greater. Poor resources, unavailability of diverse food groups puts them at risk for malnutrition.^2^ Malnutrition is a serious condition that was previously described as a condition which occurs when a person’s diet does not contain enough nutrients to meet the demands of their body.^3^ However, according to recent WHO definition 2025, it is now referred to deficiencies, excesses or imbalances in a person’s intake of energy and/or nutrients.^4^ Unfortunately Pakistan falls in a low-middle income country and food deprivation is a major challenge, especially among the low socioeconomic population, so under nutrition poses a greater risk in our population.^5^ Along with food scarcity, improper food practices such as introduction of tea/ water with biscuits between 4-6 months of age, cow milk before one-year of age , bottle feeding etc. further add upon the nutritional decline in such children.^6^ Under-nutrition also puts children at higher risk of dying from common infections and delayed recovery from minor illnesses.^5^ Pre-school age is a time when majority of the food groups should be introduced in order to prevent the adverse effects of poor eating habits in the long term.^3^ In a national survey done in 2018, it was found that only 15% of children in 6-23 months of age had adequate dietary habits.^3^ Preference to low-protein and high-carbohydrate diet, frequency of meals in a day, types of snacks taken, socioeconomic status and increased frequency of junk food consumption are among the major responsible factors having an impact on a child’s overall nutritional status.^3^,^7^

According to a National Nutrition Survey done in 2011, 31% of children less than five years of age in Pakistan are underweight.^5^ One study affirmed that various cultural backgrounds also affect the adoption of certain food items.^8^ A comparative study assessing differences in food behaviors in children from urban and rural settings of Pakistan showed that urban children have high intake of all the essential nutrients of the body.^9^ Interestingly, in a research done in Sri Lanka, it was found that the dietary diversity in toddlers was high in children who had elderly (>60 years) caregivers at home or those belonging to Muslim ethnicity.^1^ Moreover, Muslim community children had higher DDS scores as compared to Sinhalese children.^1^ Also, boys had poor dietary habits as compared to girls.^5^,^10^,^11^

Although data is available for school aged children, information regarding dietary diversity among pre-school children remains limited. Evidence also suggests that improving dietary choices among mothers positively influences the dietary patterns of their children.^12^-^15^ Therefore, this study aims to assess the types of food groups commonly consumed by preschool children as the findings will provide physicians and primary care providers with evidence-based insights to support targeted nutritional counselling and interventions for this vulnerable age group. Our objectives were:

To assess the dietary diversity among pre-school children aged 2-5 years visiting outpatient clinics in Karachi.

To evaluate the relationship between dietary diversity scores and the nutritional status of pre-school children as measured by body mass index (BMI) and mid-upper arm circumference (MUAC)

## METHODOLOGY

This was a cross-sectional study conducted at the outpatient clinics of The Indus Hospital Karachi (IHHN) carried out between a four-month period from August 2024 till November 2024.

### Ethical Approval:

It was obtained from The Institutional Review Board of The Indus Hospital and Health Network (IRB number: IHHN_IRB_2023_10_023; dated February 28, 2024*). Data confidentiality and anonymity were maintained throughout the study. There were no potential risks involved in the study*.

### Study population and Sampling technique:

Non-consecutive convenience sampling was used to recruit participants. Children aged 2-5 years visiting the outpatient department for routine immunization or minor, short-term illnesses (such as upper respiratory tract infections, common cold or fever) were eligible for participation. Children with ongoing treatment or on long-term medication for any chronic illness, or those diagnosed with congenital or systemic diseases were excluded.

### Sample size calculation:

The sample size was calculated to be 334 using the WHO sample size calculator based on an estimated prevalence of low dietary diversity of 4.4%(1) among children, with a margin of error of 2.2% and confidence interval of 95%.

### Data collection tool and procedure:

Data regarding dietary practices was collected using a structured questionnaire that was developed by the research team. The tool consisted of three main sections:

1. **Section 1 –** This section focused on child’s anthropometric data on age, gender, height, weight and MUAC.
2. **Section 2 - Included demographic and socioeconomic information such as occupation, education level, household income, religion and ethnicity as well as parents/caregivers characteristics such as age, relationship with the child etc.**
3. **Section 3- This section consisted of questions to collect dietary information of the child using a Food Frequency Questionnaire( FFQ),** a validated tool adapted from the tool developed by Sirasa et al.^1^ for pre-school children. This instrument has demonstrated good test–retest reliability (κ = 0.37–0.85; ICC = 0.29–0.83) and moderate criterion validity (ρ > 0.5 for key food groups). The adapted version follows the approach of Sirasa et al.^1^ who utilized the same FFQ structure to evaluate dietary patterns among preschool children, referencing the nine-food-group Dietary Diversity Score (DDS) method established by Rah et al.^16^ for South Asian settings. Consistent with these studies, our adaptation relied on the established construct validity of the DDS framework rather than local revalidation.

This adapted questionnaire assessed the frequency and type of food group consumed by the child over the preceding seven days, following the nine food group classification system recommended by FAO and WHO.^1^ The average daily consumption was calculated by dividing the sum by seven.

### Study variables:

- ***Dietary Diversity Score:*** It was calculated by summing the number of different food groups consumed during the recall period, yielding a score from 0 to 9.
- ***Low dietary diversity:*** DDS <3.0
- ***Medium dietary diversity:*** DDS 3.1 – 6.0
- ***High dietary diversity:*** DDS 6.1 – 9.0

***Anthropometric indicators:*** Weight was measured using a calibrated digital weighing scale and height was measured with a portable stadiometer. BMI was calculated as weight (kg) divided by height squared (m2) and interpreted using age and gender specific WHO BMI-for-age Z-scores.^17^ MUAC was measured at the mid-point between the acromion and olecranon processes using a non-stretchable MUAC tape.^18^ Nutritional status based on MUAC was categorized as follows:

**≥ 12.5 cm** normal

**11.5 - <12.5cm moderate acute malnutrition (MAM)**

**<11.5cm severe acute malnutrition (SAM)**

### Statistical analysis:

Data was analyzed in SPSS Version 26. Continuous variables were summarized as means and standard deviations, and categorical variables as frequencies and percentages. Group differences in DDS, BMI, and MUAC were assessed using independent t-tests and chi-square tests. Normality was checked with the Shapiro–Wilk test. Associations between dietary diversity and anthropometric indicators were examined using ordinal regression, adjusting for child and caregiver characteristics. A p-value <0.05 indicated statistical significance.

## RESULTS

### Sociodemographic characteristics:

A total of 334 pre-school children were included in the study, with 56.9% males and 43.1% females (Table-I). Most of the children were aged 24-35 months (47.6%), followed by 36-47 months (26.0%) and 48-60 months (26.3%). The majority (96.1%) resided in urban areas, while 3.9% visited from rural settings. Among parents, 84.1% were female respondents and 63.5% were aged 25-29 years. Most parents were not working (79.9%) while 10.5% were engaged in unskilled and 9.6% in semi-skilled or skilled occupations. More than half of the households (52.7%) had a monthly income between PKR 30,001 – 40,000, 34.1% earned **≤**30,000 PKR and 13.2% earned >40,000 PKR.

**Table-I:** Comparison of Socio-demographic Characteristics with Dietary Diversity Scores

Characteristic	Group	n	%	DDS Mean (SD)	95% CI	p-value ^A^
Total		334	100%	2.46 (0.74)	[2.38, 2.54]	
Age Group						0.208
	24–35 months	159	47.60%	2.42 (0.75)	[2.31, 2.54]	
	36–47 months	87	26.00%	2.41 (0.67)	[2.26, 2.55]	
	48–60 months	88	26.30%	2.58 (0.79)	[2.41, 2.75]	
Gender						0.016
	Male	190	56.90%	2.54^B^ (0.76)	[2.43, 2.64]	
	Female	144	43.10%	2.36 (0.71)	[2.25, 2.48]	
Residence						<0.001
	Urban	321	96.10%	2.50^B^ (0.72)	[2.42, 2.58]	
	Rural	13	3.90%	1.60 (0.68)	[1.19, 2.02]	
Parent Gender						0.512
	Female	281	84.10%	2.45 (0.72)	[2.37, 2.53]	
	Male	53	15.90%	2.52 (0.85)	[2.29, 2.76]	
Parent Age Group						0.0024
	18–24 years	44	13.20%	2.11^C^ (0.74)	[1.88, 2.33]	
	25–29 years	212	63.50%	2.50^B^ (0.73)	[2.40, 2.60]	
	30–37 years	78	23.40%	2.56^B^ (0.74)	[2.39, 2.72]	
Parent Occupation Group						0.86
	Not working	267	79.90%	2.47 (0.71)	[2.38, 2.55]	
	Semi-skilled & Skilled/Professional	32	9.60%	2.39 (0.95)	[2.05, 2.73]	
	Unskilled	35	10.50%	2.47 (0.80)	[2.20, 2.75]	
Household Income						<0.001
	≤30,000 PKR	114	34.10%	2.25^B^ (0.77)	[2.10, 2.39]	
	30,001–40,000 PKR	176	52.70%	2.52^B^ (0.66)	[2.42, 2.62]	
	>40,000 PKR	44	13.20%	2.79^B^ (0.82)	[2.54, 3.04]	
Parent Education						<0.001
	Uneducated	51	15.30%	1.83^B^ (0.64)	[1.65, 2.01]	
	Primary	91	27.20%	2.40^C^ (0.62)	[2.27, 2.53]	
	Secondary	123	36.80%	2.60^CD^ (0.74)	[2.47, 2.73]	
	Intermediate	64	19.20%	2.72^D^ (0.68)	[2.55, 2.89]	
	Graduate & Postgraduate	5	1.50%	3.31^D^ (0.77)	[2.36, 4.27]	
Religion						0.154
	Islam	317	94.90%	2.47 (0.74)	[2.39, 2.55]	
	Hinduism	3	0.90%	1.67 (0.54)	[0.32, 3.01]	
	Christianity	14	4.20%	2.36 (0.85)	[1.86, 2.85]	

A Mean DDS comparisons were made using independent t-test, ANOVA (or the Mann–Whitney U test and Kruskal–Wallis test if not normally distributed) and post-hoc procedures. BCD Different uppercase superscript letters indicate significant differences (p < 0.05) in child DDS between categories of demographic socio-economic characteristics as determined by post-hoc procedures.

Regarding parental education, 36.8% had secondary, 27.2% primary and 19.2% intermediate education. Only 1.5% were graduates or post graduates and 15.3% had no formal education. The majority of participants were Muslims (94.9%) while 4.2% were Christians and 0.9% Hindu.

### Comparison of Dietary Diversity Score (DDS) with Sociodemographic Characteristics:

The overall mean DDS was 2.46 ± 0.74 (95% CI: 2.38-2.54). Table-I. Children aged 48-60 months had the highest mean DDS (2.58 ± 0.79), followed by 36-47 months (2.41 ± 0.71) and 24- 35 months (2.42 ± 0.73) (p = 0.208). Mean DDS was significantly higher in males (2.54 ± 0.76) than females (2.36 ± 0.71) (p value = 0.016). Urban children had higher DDS (2.50 ± 0.72) than rural (1.60 ± 0.68, p <0.001). DDS did not differ significantly by parent gender (p= 0.512) or parent occupation (p=0.86). However, significant differences were observed across parental age (p=0.0024), household income (p <0.001) and education (p<0.001). The lowest DDS was among children of uneducated parents (1.83 ± 0.64) and those with income <30,000 PKR (2.25 ± 0.77), while the highest was among children of graduate/post graduate parents (3.31 ± 0.77) and household earnings >40,000 PKR (2.79 ± 0.82). Religion was not significantly associated with DDS (p=0.154).

### DDS distribution and Food Group consumption:

The Dietary Diversity Scores (DDS) in the study population ranged from 1 to 5 out of 9 (<1.5 to 5) (Table-II). DDS values of six and above were not present in the dataset. The largest proportion of children (81.44%) were classified as having low dietary diversity (DDS ≤ 3.0), while a smaller proportion (18.56%) fell into the medium dietary diversity category (DDS 3.1–6.0). The percentage distribution of food group consumption varied across individual DDS values. Among children with a DDS of 1, rice and milk were consumed by 65.63% and 46.88%, respectively, while the intake of green leafy vegetables (21.88%), meat (18.75%), and fish (3.13%) remained comparatively low. In contrast, children with a DDS of five demonstrated 100% consumption rates across several food groups, including rice, lentils, yellow fruits, eggs, chicken, and milk. When grouped by DDS category, children in the medium DDS group (3.1–6.0) exhibited higher percentages of food group consumption compared to those in the low DDS group (≤3.0). Green leafy vegetables were consumed by 77.42% in the medium DDS group compared to 54.04% in the low DDS group. Yellow fruit consumption was 93.55% in the medium group and 79.04% in the low group, while milk intake was reported at 98.39% and 84.56%, respectively. Additionally, higher consumption percentages of eggs (98.39% vs. 73.53%), chicken (88.71% vs. 70.22%), and meat (62.9% vs. 34.56%) were observed in the medium DDS group relative to the low DDS group.

**Table-II:** Percentage of children with DDS and percentage consumption of different food groups by DDS for children aged 24–60 months (n=334)

	Individual DDS^a^ (average DDS range)					DDS categories (average DDS range)	
	1 (<1.5)	2 (1.5–2.4)	3 (2.5–3.4)	4 (3.5–4.4)	5 (4.5–5.4)	Low DDS (≤ 3.0)	Medium DDS (3.1 – 6.0)
Number of Children	32	147	124	29	2	272	62
Percentage of children with DDS	9.58	44.01	37.13	8.68	0.6	81.44	18.56
	*Percentage consumption of food groups at individual DDS^a^*					*Percentage consumption of food groups at DDS categories*	
Food Groups	1 (<1.5)	2 (1.5–2.4)	3 (2.5–3.4)	4 (3.5–4.4)	5 (4.5–5.4)	Low DDS (≤ 3.0)	Medium DDS (3.1 – 6.0)
Rice	65.63	87.76	93.55	100	100	87.5	95.16
Lentils	53.13	79.59	91.13	96.55	100	80.51	93.55
Green leafy vegetables	21.88	53.74	66.94	86.21	50	54.04	77.42
Yellow fruit	46.88	77.55	91.13	100	100	79.04	93.55
Eggs	53.13	69.39	90.32	96.55	100	73.53	98.39
Fish	3.13	2.72	1.61	6.9	0	2.21	4.84
Chicken	43.75	68.71	82.26	93.1	100	70.22	88.71
Meat	18.75	32.65	46.77	68.97	50	34.56	62.9
Milk	46.88	85.03	97.58	96.55	100	84.56	98.39

### Association between Daily DDS and Nutritional Indicators:

Multivariable linear regression for association between average daily dietary diversity score (DDS) and body mass index (BMI) among children, adjusting for age group and parental education, and showed a positive association between DDS and BMI (β=0.38, 95% CI: 0.05 -0.71, p=0.025). Table-III Children aged 36-60 months had lower BMI compared to those aged 24-35 months (β= -0.71, p=0.003). Children of parents with no formal education had lower BMI (β= -0.90, p=0.019). The overall model was statistically significant (p < 0.001), explaining approximately 7.8% of the variance in BMI (R² = 0.0784).

**Table-III:** Multivariable linear regression model between Daily Dietary Diversity Score (DDS) and BMI (n=334)

Variable	Coefficient	Std. Error	p-value	95% CI
Average Daily DDS	0.3785	0.1681	0.025	[0.048, 0.709]
***Age group***				
24—35 months (ref.)				
36—60 months	-0.7066	0.2334	0.003	[-1.166, -0.248]
***Parental Education***				
***Primary (ref.)***				
Secondary	-0.2149	0.2889	0.458	[-0.784, 0.354]
Intermediate	0.2428	0.3442	0.477	[-0.434, 0.919]
Graduate & Post Graduate	-1.6058	0.9678	0.097	[-3.509, 0.299]
Uneducated	-0.8964	0.379	0.019	[-1.642, -0.151]
Constant	15.0528	0.4784	<0.001	[14.112, 15.993]

*The model is adjusted for child’s age group and parental education. P< 0.005 considered statistically significant.

In the adjusted linear regression model (Table-IV), average daily DDS was positively associated with MUAC (β =0.68, 95% CI:0.49-0.87, p < 0.001). Higher MUAC was also linked with older age (β = 0.53, p <0.001), male gender (β = 0.27, p= 0.043) and older parental age (β = 0.05, p= 0.047). Children of unskilled workers had significantly lower MUAC (β = -0.59, p=0.009), whereas higher MUAC was seen in children from middle-income households (β= 0.36, p=0.015) and with intermediate parental education (β = 0.43, p=0.044). The model explained 35% of MUAC variance (Adj. R² = 0.325).

**Table-IV:** Multivariable linear regression model assessing the association between average daily dietary diversity score (DDS) and mid-upper arm circumference (MUAC) (n=334)

Variable	Coefficient	Std. Error	p-value	95% CI
Average Daily DDS	0.68	0.095	<0.001	[0.493, 0.868]
***Age Group***				
24—35 months (ref.)				
36—60 months	0.533	0.134	<0.001	[0.270, 0.796]
***Gender***				
Female (ref.)				
Male	0.267	0.131	0.043	[0.008, 0.525]
Parent age	0.047	0.024	0.047	[0.001, 0.093]
***Parent Occupation***				
***Not working (ref.)***				
Semi-skilled & Skilled/Professional	-0.053	0.239	0.826	[-0.522, 0.417]
Unskilled	-0.59	0.225	0.009	[-1.034, -0.147]
***Income Group***				
<30,000 (ref.)				
30,001 - 40,000	0.36	0.148	0.015	[0.069, 0.651]
>40,000	0.065	0.247	0.794	[-0.422, 0.551]
***Education***				
***Primary (ref.)***				
Secondary	0.272	0.165	0.099	[-0.051, 0.596]
Intermediate	0.43	0.212	0.044	[0.012, 0.847]
Graduate+	0.627	0.573	0.274	[-0.500, 1.754]
Uneducated	-0.359	0.218	0.101	[-0.788, 0.070]
Constant	9.172	0.666	<0.001	[7.861, 10.483]

*The model is adjusted for child’s age group and gender, parental age, occupation, income, and educational status. p < 0.05 considered significant.

## DISCUSSION

Children who consume a greater number of food groups in the early years tend to have overall better health outcomes.^2^,^18^ Hence, it is important for children to have various food groups introduced into their diet during early childhood in order to support better developmental performance throughout life. In this study, it was concluded that the majority of children exhibited low DDS (i.e. <3.0) which was even lower than the parent study done in Sri Lanka.^1^ Bangladesh, whose economic status is comparable to Pakistan, also showed somewhat similar results but the scores were slightly better in studies previously done on dietary diversity in their children in both urban and rural areas.^19^,^20^ The data comprised a predominantly urban population, with fewer participants from rural areas and there was significant association between urban residence and better dietary diversity as compared to rural (p value <0.001). Similar results were found in a study conducted in China as well as in Bangladesh, Nepal and Lebanon, where children from urban areas showed better dietary diversity^21^ and a positive association between DDS and parents’ education (p value <0.001), household income ( p-value <0.001) and parent age group (p-value <0.0024), where older parents in the late 20s and mid 30s contributed to better DDS outcomes.^2^,^17^,^19^,^20^,^22^,^23^ The majority of accompanying parents were mothers; hence, their education was found to have significant association with the child’s dietary habits (<0.001). Similar to what was found in previous studies, most parents had a secondary level of education, whereas the highest DDS scores were observed among children whose parents were university graduates; however, this group represented the smallest proportion of the sample.^1^,^10^,^24^ Interestingly, cultural and religious differences were not found to have significant impact on the dietary diversity of our children which was in contrast with the parent study by Sirasa et al., where Muslim children exhibited higher DDS.^1^ This could have been because the comparison group did not have enough sample size for robust analysis. Interestingly, male children in this study were found to have higher DDS than females^25^ which might be because of the cultural preference given to male children in our society. A high DDS category was completely absent from our data which is likely attributable to the restricted sample from a majorly poor socioeconomic stratum, where access to diverse food groups is constrained.

Red meat consumption was lower among the majority of children, likely owing to its cost, however, chicken was fairly consumed. Similar findings were found in the Sinhalese population where chicken was consumed the most.^1^ Fish consumption, however, showed inconsistent findings in this study. Some people who had access to fish at minimal prices or themselves were vendors had good intake of it, whereas most of children were deprived of fish, probably owing to its cost in the market. Notably, none of the children in this study consumed soft drinks or fruit juices which is in contrast to a study done previously where high intake of soft drinks and sugary foods led to obesity in children.^8^,^26^

Overall, poor intake of fresh fruit and vegetables, legumes, meat and milk was observed in our population, similar to what was found in previous studies.^2^,^27^ Milk intake in the overall sample, however, was considerably good which might be because of being a good and easy alternative to food items. However, regarding fast food, the data showed that children were not inclined towards it probably due to its cost. Interestingly, rice was a popular food group among this study group, similar to findings in the Sri Lankan population.^1^

The results showed that better DDS is associated with improved BMI in children, which supports the hypothesis that consuming a wider variety of food groups promotes optimal growth and development. This result aligns with regional studies, such as those from Bangladesh and Sri Lanka.^1^,^19^,^20^ However this finding contrasts with a study done in China^21^, which suggested that DDS had an overall negative relationship with BMI, classifying it more as an indicator of micronutrient inadequacy than obesity risk. The divergence is particularly notable regarding age where a positive correlation was found in younger children (<6 years), while our results showed that older children in our cohort tended to have better DDS and BMIs.^21^ The positive association of DDS with higher parental age and male gender is also consistent with regional demographic determinants of dietary quality.^1^,^22^ The positive influence of higher parental age and male gender on DDS also translated into improved MUACs, strengthening the argument for shared socio-economic and cultural mechanisms influencing dietary quality and subsequent acute nutritional status within the South Asian region, showing similar trends observed in studies from Bangladesh and Sri Lanka.^1^,^18^,^20^,^22^ Acknowledging this gender disparity is crucial, as it indicates that simply increasing food availability may not resolve malnutrition; rather, targeted interventions addressing intra-household resource distribution and gender norms are necessary to ensure equitable access to diverse diets for all children.^2^,^9^,^22^

### Strengths of the study:

The study focused on the critical development period to assess the relationship between nutrition, growth and cognition. Strengths include high clinical applicability due to diverse recruitment from both Family Medicine and Pediatric Outpatient departments and a comprehensive scope that mapped the associations with BMI, MUAC and influential parental factors.

### Limitations

Limitations include poor generalizability (sample from urban slums in Karachi), potential selection and recall bias, and the use of an adapted FFQ that lacks formal validation in the Pakistani context, necessitating future psychometric assessment.

## CONCLUSION

This study highlights a concerningly low level of dietary diversity among children aged 2–5 years, with no cases of high dietary diversity observed. These findings suggest a strong association between low dietary diversity and poor socioeconomic conditions, particularly in households with limited income and lower maternal education. These results emphasize the need for targeted nutritional interventions and public health policies aimed at improving dietary practices in early childhood, particularly among underserved populations. Educating caregivers, especially mothers, and increasing access to diverse and affordable food options may play a critical role in addressing early childhood malnutrition in similar low-resource settings. It also requires immediate action to address the gender disparity in food distribution to ensure equitable nutritional access to all children and finally, strengthening future research by obtaining a more balanced representation of rural data to better inform policy.

### Author`s contribution:

**UK:** Study concept and design, designed the questionnaire, literature search , data collection and manuscript writing.

**HA:** Study design, questionnaire design, data interpretation, and critical manuscript review.

**AR:** Contributed to the data interpretation, analysis of results and critical review.

All authors have read the final version and are responsible and accountable for the accuracy and integrity of the work.
